# Food Finding Test without Deprivation: A Sensorial Paradigm Sensitive to Sex, Genotype, and Isolation Shows Signatures of Derangements in Old Mice with Alzheimer’s Disease Pathology and Normal Aging

**DOI:** 10.3390/brainsci14030288

**Published:** 2024-03-18

**Authors:** Daniela Marín-Pardo, Lydia Giménez-Llort

**Affiliations:** 1 Institut de Neurociències, Universitat Autònoma de Barcelona, 08193 Barcelona, Spain; daniela.marin@autonoma.cat; 2 Department of Psychiatry and Forensic Medicine, School of Medicine, Universitat Autònoma de Barcelona, 08193 Barcelona, Spain

**Keywords:** neuroethology, 3xTg-AD mice, behavioral neuroscience, methods, sniffing loss, aging, Alzheimer’s disease, animal models, ethogram

## Abstract

The Food Finding Test (FFT) olfactory paradigm without overnight food deprivation examined olfaction in aged (16-months-old) animals. Ethograms of three goal-directed behaviors towards hidden food (sniffing, finding and eating) elicited in male and female 3xTg-AD mice for Alzheimer’s disease (AD) and their age-matched C57BL/6 wild-type counterparts with normal aging were meticulously analyzed with the support of video recordings. The new FFT protocol elicited longer ethograms than previously reported with the standard deprivation protocol. However, it was sensitive when identifying genotype- and sex-dependent olfactory signatures for the temporal patterns of slow sniffing, finding, and eating in AD and males, but it had a striking consistency in females. The impact of forced social isolation was studied and it was found to exert sex-dependent modifications of the ethogram, mostly in males. Still, in both sexes, a functional derangement was detected since the internal correlations among the behaviors decreased or were lost under isolated conditions. In conclusion, the new paradigm without overnight deprivation was sensitive to sex (males), genotype (AD), and social context (isolation-dependent changes) in its ethogram and functional correlation. At the translational level, it is a warning about the impact of isolation in the advanced stages of the disease, paying notable attention to the male sex.

## 1. Introduction

Our perception and understanding of the world throughout our lifespan rely heavily on our sensory systems. As we age, the timing and intensity of the decline in sensory functions becomes crucial, influencing our capacity to maintain a high-quality sensory experience. This, in turn, has lasting effects on cognition, self-esteem, habits, and lifestyle. Due to the vulnerability and potential disability caused by sensory deficits, older individuals are at risk of experiencing an overall decline in their well-being on biological, psychological, and social fronts [1]. Older individuals with sensory impairments experience increased levels of biological, psychological, and social challenges [2]. Sensory deficits have been suggested as an early sign of the prodromal stages of Alzheimer’s disease among older people [3,4]. The comprehensive and thorough evaluation of patients with neurodegenerative disorders plays a fundamental role in identifying etiology, pathogenesis, differential diagnosis, uncovering and assessing risk factors, and potential therapeutic options [5].

Recent studies have even indicated that anosmia, the loss of the sense of smell, can predict a higher 5-year mortality risk compared to cardiovascular disease [6]. The correlation between odor identification dysfunction and cognitive decline is also gaining attention, with olfactory dysfunction serving as a marker of cognitive decline for various neurological and neurodegenerative conditions [7]. A connection between olfactory impairments, specifically the loss of the sense of smell, and Alzheimer’s disease is a topic of growing interest. Thus, multiple research endeavors have explored the relationship between anosmia and Alzheimer’s disease. Approximately 90% of individuals with Alzheimer’s disease exhibit some form of olfactory impairment [8]. Additionally, this loss has been noted during the transition from normal aging to dementia, particularly in cases of mild cognitive impairment. Earlier research suggests that olfactory impairment may signal an increased likelihood of dementia in individuals with mild cognitive impairment [9]. Although further investigation is required to uncover the precise mechanisms underlying this connection, the presence of toxic proteins like beta-amyloid and tau in the brain, neuropathological hallmarks of Alzheimer’s, has also been associated with the loss of the sense of smell [10]. Therefore, olfactory impairments could potentially offer a valuable tool for the early diagnosis of Alzheimer’s disease [3]. On the other hand, conducting preliminary diagnostic screenings through olfactory testing could facilitate the prompt implementation of preventive measures, thereby enhancing cognition, brain health, and mental well-being [11].

At the translational level, in this brief report, we studied the olfactory signatures in male and female mice with normal and neurodegenerative aging associated with the advanced stages of Alzheimer’s disease (AD), as well as the effects of forced social isolation. For this purpose, we used 3xTg-AD mice [12] and age-matched non-transgenic mice, both with a C57BL/6J genetic background. The 3xTgAD mouse is a genetic model of AD that presents not only AD cognitive dysfunction but also a striking phenotype which models some “behavioral and psychological symptoms of dementia” (BPSD), including neuropsychiatric symptoms such as anxiety, apathy, and depression-like behaviors [13].

One of the pioneering tests to evaluate smell in mice was the buried or hidden cookie test (HCT), an olfactory ability assessment that evaluates food-seeking behavior in mice [14]. The test involves hiding a cookie within bedding material and observing the mice as they locate the cookie based on olfactory cues. In the present work, we chose to use a more ethological and naturalistic test referred to as the Food Finding Test (FFT) [15,16]. The FFT is a neuroethological assessment of olfactory function and related behaviors in mice. The Food Finding Test considers species-specific behavioral traits and ethological responses, unlike the Hidden Cookie Test. Both tests, the HCT and the FFT, are methods used to assess olfactory function, providing insights into olfaction and its implications on behavior. However, they differ in methodology and focus. The Hidden Cookie Test focuses on food-seeking behavior to specifically target olfactory ability, whereas the Food Finding Test offers a broader assessment of olfactory function within a neuroethological framework. In our research, we use the FFT to assess olfactory function throughout the lifespan, particularly in the context of normal aging and Alzheimer’s disease related pathological aging, whilst also considering the effects of extrinsic factors such as social environment (mainly due to the impact of social isolation).

## 2. Materials and Methods

### 2.1. Animals

A total number of sixty-six 16-month-old male and female homozygous 3xTg-AD (*n* = 38, 20 male and 18 female) and non-transgenic (NTg, *n* = 29, 14 male and 15 female) mice on a C57BL/6J background (after embryonic transfer and backcrossing at least 10 generations) established in the Universitat Autònoma de Barcelona [16] were used in this study. The 3xTg-AD mice harboring transgenes were genetically engineered at the University of California Irvine, as previously described [12]. Animals of the same genotype and sex were grown and maintained in groups of three to four mice per cage (Macrolon, 35 cm × 35 cm × 25 cm), which was filled with 5 cm of clean wood cuttings (Ecopure, Chips6, DateSand, Stockport, UK; Uniform cross-cut wood granules with 2.8–1.0 mm chip size) and nesting materials (Kleenex, Art: 08834060, 21 cm × 20 cm, White). The animals were maintained under standard laboratory conditions with food and water ad lib, 22 ± 2 °C, 12-h light/dark cycle with lights on at 8:00 a.m., and a relative humidity of 50–60%.

### 2.2. Social Conditions

At 13 months of age, half of the animals were isolated and the other half remained under standard group housed conditions until their behavioral assessment at 16 months of age, corresponding to normal aging (in the NTg mice) and very advanced stages of the disease in the 3xTg-AD mice. Thus, the experimental groups were as follows: 3xTg-AD (*n* = 38, 20 male (10 group housing standard conditions, 10 Isolated) and 18 female (7 group housing standard conditions, 10 isolated) and non-transgenic (NTg, *n* = 28, 14 male (8 group housing standard conditions, 6 Isolated) and 15 female (7 group housing standard conditions, 8 Isolated). In all cases, the standard home cages were covered with a metallic grid allowing for the perception of olfactory and auditory stimuli from the rest of the colony.

### 2.3. Physical Status and Behavioral Assessment

#### 2.3.1. Physical Status

At 16 months of age, the body weight of animals was measured before the FFT behavioral paradigm as an indicator of their health status.

#### 2.3.2. The Food Finding Test of Olfactory Ability without Food Deprivation

To investigate olfactory function in old animals and advanced stages of AD disease, the Food Finding Test (FFT) olfactory paradigm [16] was used without food deprivation. In the present work, we first validate the ability of this modified protocol to elicit the different components of the Food Finding test’s goal-directed ethogram. The test was performed under dim white light (20 lx) during the light phase of the light/dark cycle (from 10:00 to 11:00 a.m.) using a novel cage (50 cm × 22 cm × 14 cm) with 1 cm of bedding. Eight food pellets (45 mg of standard food pellets) were placed in the central zone of the cage and covered by 1 cm of wood chip bedding. The mouse was placed in a corner of the cage, facing the walls, and its behavior was observed. The latencies of three goal-directed behaviors toward hidden food were recorded, namely, sniffing—when the nose makes direct contact with a surface and the vibrissae/whiskers are in maximum extension and are in contact with the surface in the same way [17]; finding (digging)—“finding a food pellet” was defined as digging, touching, and holding the pellet in the front paws for more than 3 s; and eating the hidden food—holding the pellet in the front paws for more than 3 s of continuous eating. The beddings were renewed between each mouse.

The behavioral assessments were performed using direct observation and video recordings (ViewPoint Behavior Technology, Lion, France) in a counterbalanced manner. The observer was blind to the genotype. A complementary retrospective analysis was conducted. All procedures followed the Spanish legislation on the “Protection of Animals Used for Experimental and Other Scientific Purposes” and the EU Directive (2010/63/UE) on this subject. The study complies with the ARRIVE guidelines developed by the NC3Rs and aims to reduce the number of animals used [18].

### 2.4. Statistical Analysis

The results are expressed as mean ± SEM. SPSS 20.0 software was used. A 2 × 2 ×2 factorial design was used to analyze the effects of (G) genotype, (S) sex, and (ISO) isolation factors. The differences were studied through multivariate general linear model analysis and post hoc Duncan’s test (multiple comparisons). In all the tests, *p* < 0.05 was considered statistically significant.

## 3. Results

### 3.1. Physical Status

According to Table 1, the genotype effect led to a higher body weight in male NTg mice. In contrast, genotype variances among females were associated with decreased weight in isolated animals [S*, F (1, 58) = 5.309, *p* < 0.05; G**, F (1, 58) = 7.140, *p* < 0.01)].

### 3.2. Elicitation of Olfactory Signatures despite the Lack of Overnight Food Deprivation

The ethogram of goal-directed behaviors, sniffing, finding, and eating, was preserved despite the lack of a food deprivation protocol. However, the temporal window to observe this sequence of behaviors exhibited a 3- (control mice) to 10-fold (3xTg-AD mice) amplification compared to the temporal window in our FFT protocol with food deprivation [16]. Thus, in our previous work, the scale range had a maximum of 1100 units of time, whereas in the current work, the scale is defined in a maximum range of 3500 units.

**Figure 1:**
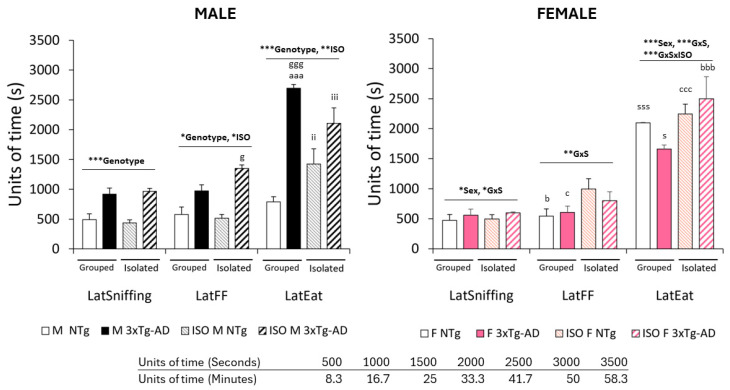
Genotype, sex, and isolation effects in the FFT in 16-month-old male and female NTg and 3xTg-AD mice. Results are expressed as mean ± SEM. Latencies in the Food Finding Test: LatSniffing, latency to sniff the hidden pellet; LatFF, latency to find the food (hidden pellet); LatEat, latency to eat the pellet. Statistics: Factorial analysis of variance 2 × 2 × 2: Effects of genotype (G), sex (S), isolation (ISO) and their interactions, * *p* < 0.05, ** *p* < 0.01, *** *p* < 0.001 vs. their respective control group. Post-hoc analysis: genotype, ^g^ *p* < 0.05 and ^ggg^ *p* < 0.001; genotype × isolation, ^aaa^ *p* < 0.001; isolation, ^ii^ *p* < 0.01 and ^iii^ *p* < 0.001; sex, ^s^ *p* < 0.05 and ^sss^ *p* < 0.001; genotype × sex, ^b^ *p* < 0.05 and ^bbb^ *p* < 0.001; sex × isolation, ^c^ *p* < 0.05; ^ccc^ *p* < 0.001.

As indicated in Figure 1 and depicted in the next sections, the FFT paradigm investigating the olfactory function in normal and AD-pathological aging elicited sex-, genotype- and isolation-dependent olfactory signatures, with alterations being enhanced in male sex.

The three goal-directed behaviors elicited statistically significant genotype differences. However, the effects were exerted in different magnitudes, with eating [Lat Eat, genotype ***, F (1, 58) = 22.586, *p* < 0.001] and sniffing [Lat Sniffing, genotype ***, F (1, 58) = 11.688, *p* < 0.001] showing the maximum effect, and finding [Lat FF, genotype *, F (1, 58) = 5.171, *p* < 0.05] a moderate genotype effect. The effect size to indicate how meaningful the relationship between these variables was further analyzed by means of behavioral correlates, as indicated below.

This gradient was also observed in the effect of sex factor, but not in all behaviors. Thus, a maximum effect of sex was shown in eating [Lat Eating, Sex***, F (1, 58) = 11.415, *p* < 0.001] and a moderate effect on sniffing [Lat Sniffing, Sex*, F (1, 58) = 4.190, *p* < 0.05], but not on finding [Lat FF, n.s., F (1, 58) = 0.967, *p* = 0.329]. This resulted in genotype × sex interaction effects seen in the three behaviors, with maximum, strong and moderate effects in eating, [LatEat, G × S***, F (1, 58) = 28.254, *p* < 0.001], finding [LatFF, G × S**, F (1, 58) = 7.859, *p* < 0.01], and sniffing [LatSniffing, G × S*, F (1, 58) = 5.222, *p* < 0.026], respectively.

Overall, the eating behavior was the one to exhibit the highest sensitivity to intrinsic factors (genotype and sex), followed by sniffing. The 2.5-fold increase in the latency of eating, compared to previous behaviors (sniffing and finding) in the 3xTg-AD mice, suggests that accumulative effects in latency scores (that is, a delay in one behavior increases the latency of appearance of the subsequent behavior) can be discarded. To further analyze this aspect, the time delays or interval of time between two consecutive behaviors were also calculated (see below).

The effects of isolation emerged in the latencies to find the food with a moderate effect [Lat FF, ISO*, F (1, 58) = 3.932, *p* < 0.05; but not in the Lat Sniffing, n.s., F (1, 58) = 0.030, *p* = 0.862] that increased to its maximum in the latency of eating it [Lat Eating, ISO***, F (1, 58) = 7.185, *p* < 0.001]. In this last goal-directed behavior, genotype and sex factors interacted with isolation [Lat Eating, G × S × I***, F (1, 58) = 16.037, *p* < 0.001; but not in the Latency of Sniffing, n.s., F (1, 58) = 0.067, *p* = 0.797; Lat FF, n.s., F (1, 86) = 2.075, *p* = 0.155] eliciting different behavioral signatures in each of the four experimental groups.

### 3.3. Time Delays and FFT Behavioral Correlates

The time delays between goal-directed behaviors in the FFT was analyzed. The results per each sex and factorial analysis is depicted in Table 1. Significant genotype-dependent differences between 3xTg-AD male mice and NTg male mice were reported in the food-finding delay and the total time between the start of the ethogram (sniffing) and its end (eating).

**Figure 2:**
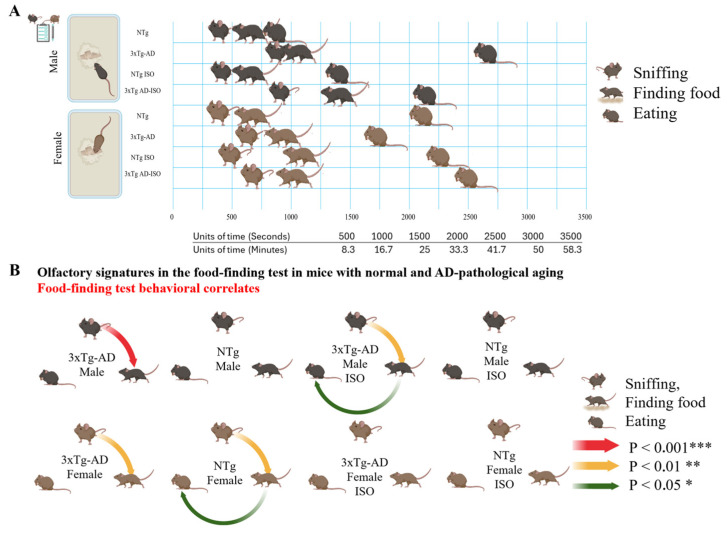
Sex- and genotype-dependent olfactory signatures in mice with normal and AD-pathological aging in the FFT without overnight food deprivation. (**A**) Experimental design and ethogram tablature for “sniffing, finding, and eating the hidden food pellet” by 16-month-old male and female NTg and 3xTg-AD mice and respective isolated groups. (**B**) Meaningful correlation analysis in the FFT: Graphical representation of the significant Pearson r correlations * *p* < 0.05, ** *p* < 0.01, *** *p* < 0.001 between the three olfactory actions, all of them positive. The figure was created with BioRender.com, accessed on 20 December 2023.

Figure 2A illustrates the olfactory signatures of 16-month-old animals with normal and AD-pathological aging elicited in the FFT, despite the lack of overnight food deprivation, and under the effects of intrinsic (genotype and sex) and extrinsic (isolation) factors. In addition, as shown in Figure 2B, meaningful correlation analysis revealed that sniffing and finding exhibited a strong and predictable relationship in 3xTg-AD males (*p* < 0.001) and females (*p* < 0.01). Two other groups, in particular NTg females and ISO Male 3xTg-AD mice also showed positive correlations between the latencies of subsequent behaviors (sniffing and finding; finding and eating).

## 4. Discussion

The FFT [15], based on species-specific behavioral traits and their ethological responses, facilitates a neuroethological assessment of olfactory function and related behaviors. It presents an easy-to-use assessment tool that can be used in any animal department without needing to purchase additional items such as the odors commonly used in other olfactory tests, and also provides the opportunity to run experimental designs without experimental odor carryover effects [14,19,20,21]. On the other hand, the incorporation of ethologically salient stimuli, such as the search of food, is particularly interesting not only for evaluating olfactory function in health and disease throughout the life cycle, but also for the associated cognitive and emotional performance, and in assessing preventive/therapeutic interventions and the impact of risk factors and hazards. Thus, we recently proposed its use as a sensorial paradigm to unveil genotype and sex differences in 12-month-old 3xTg-AD mice in a C57BL/6 genetic background as compared to wildtype mice with normal aging [16]. In the 3xTg-AD mice, this age corresponds to the advanced stages of the disease with beta-amyloid brain pathology, whereas it is considered middle age in the wildtype mice [22]. In this animal model, more advanced neuropathological stages of AD disease are characterized by concomitant beta-amyloid and tau pathologies, but there is also a strong survival bias [15] and frailty [23]. Therefore, in the present work, we studied the capacity of this test to detect genotype and sex differences in 16-month-old animals when the paradigm is administered without previous overnight food deprivation. In addition, we studied whether their olfactory signatures would be modified by forced isolation, as in our previous work in which we could observe naturalistic isolation due to the death of male cage mates [16].

The most important finding is that the current Food Finding Test paradigm for older animals elicited distinct olfactory signatures despite the lack of overnight food deprivation. As expected, genotype was the most important factor, as significant statistical differences were found in the three goal-directed behaviors, with delayed olfactory behavioral responses in the 3xTg-AD mice as compared to mice with normal aging. Overall, eating behavior was notable as it showed a considerable delay and was sensitive to genotype and sex factors, whereas sniffing and finding were elicited in a quite consecutive manner, a sequence that was found enhanced in 3xTg-AD mice under isolation. Interestingly, sex appeared to be a relevant factor since these effects were mostly due to the behavioral ethogram elicited in males, whereas females showed a strong consistency in the similarity of their latencies in all the three goal-directed behaviors as compared to those of their non-transgenic counterparts with normal aging.

Compared to the previous protocol at 12 months of age (Supplementary Table S1), the ethograms of both male and female wildtype mice with the gold-standard C57BL/6 genetic background exhibited a 2-fold increase in recorded latencies. Several intrinsic factors could account for these results. The first consideration is the age factor, as behavioral responses in 16-month-old animals tend to be slower than those observed in their middle-aged counterparts. On the other hand, the lack of overnight food deprivation in the current paradigm for old animals could also reduce the energetic demands of animals, and therefore, reduce the elicitation of fast goal (food)-directed ethograms. However, male NTg mice had lower weights than their transgenic counterparts but showed faster patterns than them. In addition, whilst the latencies of sniffing and finding were similar between males and females, the latency of eating was significantly increased in 16-month-old females, suggesting this final action (eating) was not driven by quick energy production. In addition, the sex factor interaction with genotype and/or isolation also suggests complex interactions in the elicitation of this behavioral ethogram.

Noteworthy, in the current work, the ethograms of 3xTg-AD mice differed from those reported in their congeners at 12 months of age in which fast signatures were observed [16]. The age/stage-dependent increase of weight in this animal model, mostly in males, could explain the strong delays observed in this sex. However, we consider that the advance in their neuropathology status could explain a different coping-stress strategy when confronting a new scenario, being a faster response in beta-amyloid stages but slower when the brain damage severity associated to beta- and tau-pathologies increases. These differences would agree with age/stage-dependent fast/slow responses we have recently reported in the T-maze for this [23] and other [24] AD-animal models, and those previously described in mice models with accelerated aging [25]. In both cases, these factors would explain the ethograms in male sex, whereas the similarities in females would agree with behavioral convergence we have also described in the old to end-of-life stages in this sex [23]. Therefore, these results support the understanding that male and female sexes can be considered distinct natural scenarios for studying the influence of biological, psychological, and social factors on health and illness across the lifespan, as well as the interconnected dynamics affecting homeostatic networks.

While the comparison between 3xTg-AD and NTg animals revealed substantial genotype disparities across all three olfactory actions, the current protocol also was sensitive to isolation. Thus, in contrast to previous work in which isolation was only naturally occurring in male 3xTg-AD cases [16] in the current work, in all experimental groups half of the animals were submitted (forced) to a period of isolation. While social isolation had a relatively modest effect on response latencies, it had distinctive outcomes for males and females. In females, olfactory signatures remained relatively preserved, suggesting a degree of resilience in the face of isolation. However, the situation was quite different for males, to which social isolation disrupted the established patterns of olfactory behavior. These results highlight the importance of considering gender in the study of olfaction and its susceptibility to external factors. The results also showed that derangements of the FFT-induced olfactory ethograms were also observed in female 3xTg-AD mice and their non-transgenic counterparts. Therefore, here we report for the first-time sex-specific differences within the olfactory domain, a finding undocumented in previous studies conducted under naturalistic isolation conditions. Isolated males exhibited slower responses, contrasting with the faster reactions observed in isolated females. This novel finding points at the complex interplay between sex influences and olfactory-related behaviors under positive/negative social conditions.

Meaningful correlation analysis between goal-directed behaviors provided further evidence of the strong and predictable relationship between sniffing and finding in some groups. The lack of significant correlations in male NTg mice highlights genotype-specific differences in the olfactory behavior ethogram. With regards to the impact of social condition on olfactory behaviors, sniff latency emerged as a predictive variable for finding food in isolated male 3xTg-AD mice, yet this correlation did not extend to the act of eating the food. Social isolation led to the disruption of functional correlations among goal-directed behaviors in the other groups, especially pronounced in males, indicating that the interplay between different behaviors is particularly sensitive to the effects of isolation. The loss of internal correlation among behaviors in the context of isolation adds another layer of complexity to the study of olfactory behavior, highlighting the interconnectedness of different behaviors and also revealing how external factors like social isolation can disrupt these connections. On the other hand, the complexity of their sensory–motor components demand further research on the effect size of variables and differences between groups with respect to other behavioral dimensions. Thus, in the current paradigm it is assumed that food-finding and eating are primarily olfactory behaviors, but the contribution of appetite and consummatory behaviors cannot be excluded. Conversely, bulbectomized (totally anosmic) rodents show robust sniffing. As with literature on measuring olfactory detection and discrimination in rodents [20,26], methods like habitation/dishabituation or operant methods should be used to validate the current method.

## 5. Conclusions

This behavioral research underscores the importance of considering intrinsic (genotype and sex) and extrinsic (isolation) factors when studying olfactory function in the context of aging and AD. Moreover, it demonstrates that this novel paradigm without overnight food deprivation adapted to old animals is sensitive to both intrinsic and extrinsic factors. The elicitation of genotype, sex, and isolation-dependent olfactory signatures contributes to the understanding of the complex aging and AD scenarios, underscores the potential of olfactory behavior as a valuable diagnostic tool, and provides a translational tool to design and assess tailored preventive/therapeutic strategies. Together with our previous work, these findings provide valuable insights into the effects of social isolation, especially in advanced stages of AD disease, with special concern on its impact on males, highlighting the need for specific attention in understanding and addressing these sex-specific challenges.

In summary, the integration of genotype-dependent olfactory signatures, sex-specific responses to social isolation, and the disruption of functional correlations underlines the multifaceted nature of olfactory research in aging and AD. As we explore the complexities of olfaction in AD, it becomes increasingly clear that a comprehensive understanding of these interactions is crucial.

## Figures and Tables

**Table 1:** Time delays between goal-directed behaviors in the FFT without overnight food deprivation in 16-month-old male and female NTg and 3xTg-AD mice in group housing conditions or under forced isolation.

		NTg	ISO NTg	3xTg-AD	ISO 3xTg-AD
		Mean ± SEM	Mean ± SEM	Mean ± SEM	Mean ± SEM
Body Weight	Males	46.90 ± 2.18	44.53 ± 1.52	33.60 ± 1.08 ***	30.97 ± 0.50 ***
Factor: G**, S*	Females	30.23 ± 1.77	30.41 ± 1.24	27.89 ± 0.97	24.49 ± 0.76 **
Sniffing—Finding	Males	130.2 ± 83.5	54.3 ± 34.0	80.4 ± 63.4	382.2 ± 192.4
Factor: **ISO	Females	70.0 ± 35.2	498.8 ± 183.4	48.7 ± 42.2	200.4 ± 152.7
Finding—Eating	Males	552.7 ± 112.4	1720.0 ± 142.0	906.6 ± 274.5	757.4 ± 240.5
Factor: **S	Females	1556.6 ± 289.6	1253.1 ± 255.2	1055.3 ± 233.9	1697.0 ± 319.9
Sniffing—Eating	Males	422.5 ± 111.0	1774.3 ± 133.9	987.0 ± 264.0	1139.6 ± 256.0
Factor: **G × S	Females	1626.6 ± 292.5	1751.9 ± 198.2	1104.0 ± 241.1	1897.4 ± 358.9

Note: Statistics: Factorial analysis of variance 2 × 2 × 2 and post hoc Duncan’s test. Effects of genotype (G), sex (S), isolation (ISO) factors and their interactions, * *p* < 0.05, ** *p* < 0.01, *** *p* < 0.001 vs. their respective control group. Male NTg (*n* = 6), ISO Male NTg (*n* = 8), Male 3xTg-AD (*n* = 10), ISO Male 3xTg-AD (*n* = 10), Female NTg (*n* = 7), ISO Female NTg (*n* = 6), Female 3xTg-AD (*n* = 10), ISO Female 3xTg-AD (*n* = 7).

## Data Availability

The data presented in this study are available on request from the corresponding author. The raw data supporting the conclusions of this article will be made available by the authors.

## Supplementary Materials

The following supporting information can be downloaded at: https://www.mdpi.com/article/10.3390/brainsci14030288/s1, Table S1. Food finding test in 3xTg-AD and NTg mice under naturalistic and forced isolation.
