# Supplementary material for: Food Finding Test without Deprivation: A Sensorial Paradigm Sensitive to Sex, Genotype, and Isolation Shows Signatures of Derangements in Old Mice with Alzheimer’s Disease Pathology and Normal Aging
Source: Brain Sci. 2024 Mar 18;14(3):288. doi: 10.3390/brainsci14030288 (PMC10968469; doi:10.3390/brainsci14030288)
Supplement: Supplementary file 1 [file brainsci-14-00288-s001.zip › brainsci-2866916-supplementary.pdf]

brain sciences

Table S1. Food finding test in 3xTg-AD and NTg mice under naturalistic and forced isolation.

Naturalistic Social Isolation [14] 12-month-old with overnight food deprivation

	NTg Male (n=24)			3xTg-AD Male (n=15)			3xTg AD-ISO Male (n=7)		
	Mean		± SEM	Mean		± SEM	Mean		± SEM
Food finding test									
Lat of sniffing (s)	251	±	63.8	135.7	±	16.2	85.1	±	14.5
Lat of finding food (s)	424.2	±	57.9	152.4	±	15.7	111.8	±	17.1
Lat of eating (s)	912.4	±	121	193	±	17.7	138.1	±	23.6

Forced Social Isolation (current work) – 16-month-old without overnight food deprivation

	NTg Male (n=6)			NTg-ISO Male (n=8)			3xTg-AD Male (n=10)			3xTg AD-ISO Male (n=10)		
	Mean		± SEM	Mean		± SEM	Mean		± SEM	Mean		± SEM
Food finding test												
Lat of sniffing (s)	436.5	±	50.8	967.3	±	212	919.4	±	102.5	491.7	±	100.1
Lat of finding food (s)	516.9	±	60.1	1349.5	±	305.6	973.7	±	103.3	578.5	±	124.3
Lat of eating (s)	1423.5	±	258.4	2106.5	±	295.2	2693.7	±	63.9	47.5	±	3.5
